# Electrical Characterization of Gold-DNA-Gold Structures in Presence of an External Magnetic Field by Means of I–V Curve Analysis

**DOI:** 10.3390/s120303578

**Published:** 2012-03-14

**Authors:** Nadia Mahmoudi Khatir, Seyedeh Maryam Banihashemian, Vengadesh Periasamy, Richard Ritikos, Wan Haliza Abd Majid, Saadah Abdul Rahman

**Affiliations:** Low Dimensional Materials Research Centre, Department of Physics, University of Malaya, 50603, Kuala Lumpur, Malaysia; E-Mails: vengadeshp@um.edu.my (V.P.); richardritikos@gmail.com (R.R.); q3haliza@um.edu.my (W.H.A.M.); saadah@um.edu.my (S.A.R.)

**Keywords:** Metal-DNA-Metal (MDM), magnetic field, magnetic sensor, Schottky diode

## Abstract

This work presents an experimental study of gold-DNA-gold structures in the presence and absence of external magnetic fields with strengths less than 1,200.00 mT. The DNA strands, extracted by standard method were used to fabricate a Metal-DNA-Metal (MDM) structure. Its electric behavior when subjected to a magnetic field was studied through its current-voltage (I–V) curve. Acquisition of the I–V curve demonstrated that DNA as a semiconductor exhibits diode behavior in the MDM structure. The current *versus* magnetic field strength followed a decreasing trend because of a diminished mobility in the presence of a low magnetic field. This made clear that an externally imposed magnetic field would boost resistance of the MDM structure up to 1,000.00 mT and for higher magnetic field strengths we can observe an increase in potential barrier in MDM junction. The magnetic sensitivity indicates the promise of using MDM structures as potential magnetic sensors.

## 1. Introduction

For several decades research on DNA material and DNA-based devices has attracted huge attention. In a great deal of research, electron transfer through molecular wires and DNA strands has been modelled as donor-bridge-acceptor systems due to the analogy between them. DNA as a molecular wire plays a key role in exhibition of nonlinear behaviors in I–V characteristic curves [1–6]. Once DNA is sandwiched between metal layers, these wires enable charge transport phenomena as a rectifier or transistor or switch to take place [5–8]. The important and interesting issue in DNA base device is their semiconductive behavior [9,10] or response in the presence of external electric and magnetic fields [11]. Some semiconductors, such as silicon, are fabricated under high temperature conditions. The provision of tunable conditions for these materials with the purpose of their utility as spintronic devices is difficult. But, there are other types of semiconductor like organic semiconductors whose preparation does not involve high temperatures. They can be synthesized at much moderate temperatures with low pollution and reduced toxic effects. Furthermore, their high flexibility guarantees tunable electronic properties. The long coherence time of bio-semiconductors is a suitable feature for fabrication of coupling spin-orbit devices as a spintronic component [12]. Spin transport in molecular systems, as a branch of electrotransitions, is of special interest but lacks thorough experimental investigation [13]. Several works have been conducted concerning current transport in DNA structures in magnetic fields but many of them are theoretical studies and still need more investigation as experimental research. In this regard, Petrove *et al.* have theoretically studied the influence of an external magnetic field upon a molecular wire [4,11] and in 2002, Dawei *et al*. reported the magnetic resistance of G4-DNA in a molecular device [14].

In this work, we demonstrate an electrical behavior of a MDM structure in an external magnetic field. The MDM structure as a back-to-back diode in gold-DNA-gold structure shows low threshold voltage bias in forward and large in reverse. This diode, under the influence of an external magnetic field, acts as a magnetic diode. The potential barrier between DNA and gold is calculated to be 0.878 eV based on its I–V curve and Schottky’s rule. An external perpendicular magnetic field reduces the rate of charge transport and current through DNA strands. Based on the results, the measurements ascertain a good relationship between external magnetic field and current. The authors intend to exploit such magnetic sensitivity behavior of MDM structures in bioengineering studies and nanoelectronic devices.

## 2. Experimental Section

### 2.1. Materials

DNA molecules with sequence A (22%), T (20%), G (35%), C (23%) from *Boesenbergia rotunda* plant were extracted using the common facilities available in most laboratories [15]. A p-type Si wafer (orientation <100>) possessing a resistivity of 1 to 10–20 Ω-cm (MEMC Electronic Materials) together with a 1,000 nm thick SiO_2_ layer was used as the substrate. The chromium and gold wire (Kurt J. Lesker Company) were used in evaporation and magnetron sputtering technique had a purity of 99.999%. Other necessary chemicals (NH_3_, H_2_O_2_, HF, HCl and acetone) were supplied by Sigma Aldrich and were used without further purification. Finally, the deionized water used in the experiments was obtained from a Barnstead (Nanopure II) water deionizing system available in the laboratory.

### 2.2. Fabrication of Chip

**Figure 1.**
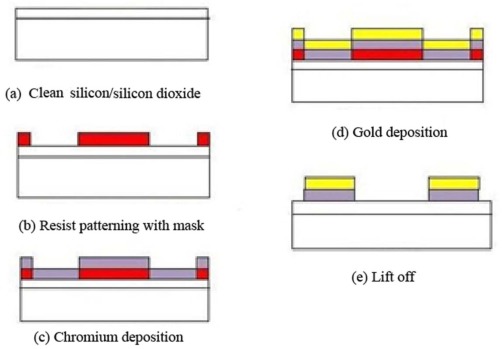
Schematic diagram showing the sample preparation. (**a**) The sample preparation. (RCA); (**b**) The photoresist deposition and UV-exposure through the mask; (**c**) Chorumum deposition; (**d**) Gold deposition; (**e**) Lift off process.

After cleaning wafers according to the standard method (RCA) and drying by nitrogen gas (as shown in Figure 1(a)), the photoresist AZl512 was deposited using spin coating and UV-lithography through the designed mask (as shown in Figure 1(b)). After this process, chromium and then gold deposition on silicon substrate of 90 and 150 nm respectively was achieved by DC magnetron sputtering and thermal evaporation (as in Figure 1(c,d)). A lift-off process is followed as the very final part of an experiment (see Figure 1(e)).

### 2.3. Measurements

DNA solution was first diluted to a suitable concentration of 0.01 mg/mL and allowed to flow along the gap between gold electrodes using a micro-syringe. Once a 70–100 μL drop of the DNA solution is deposited in the gap under influence of an external 200 kHz electric field (a 12 volt AC supply) for few second, the probability of DNA-gold contact rises. Then, a 12 volt DC supply is connected to the electrodes that align DNA strands parallel to the electric field.

**Figure 2.**
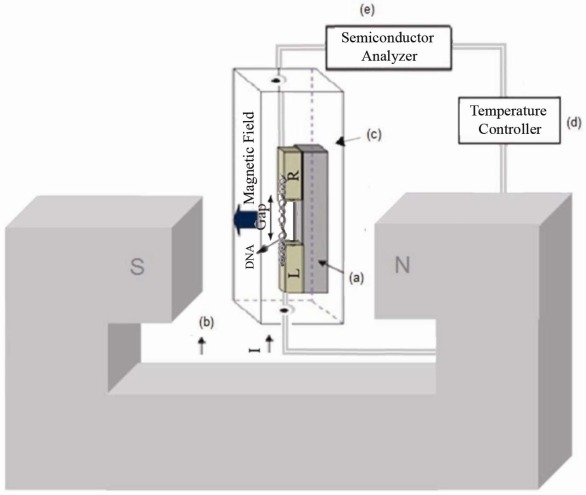
Two electrodes L and R (L = left, R = right) with an insulator gap in the gold-DNA-gold structure in the presence of external magnetic field is placed inside cryostat connected to temperature controller, semiconductor analyzer. (**a**) MDM; (**b**) Electromagnet; (**c**) Cryostat; (**d**) Temperature controller; (**e**) Semiconductor analyzer.

I–V characterization of MDM in the presence and absence of magnetic field, generated by an Electromagnet 3472-50, in dark conditions (inside a cryostat with light protection) was achieved using a semiconductor analyzer (SMU-236, Keithly) at different temperatures using a temperature controller Lakeshore-331 (as depicted in Figure 2).

## 3. Results and Discussion

Figure 2 illustrates the setup for measuring the gold-DNA-gold in the presence of a perpendicular magnetic field. The sample was placed in a cryostat under the magnetic field that generated by electromagnet while connected to a semiconductor analyzer, and a temperature controller.

**Figure 3.**
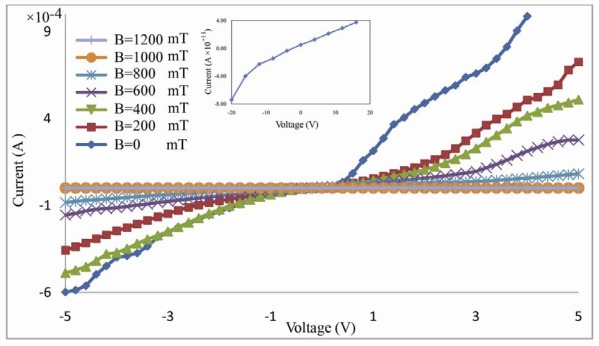
The I–V curve for gold-DNA-gold structure in presence of various magnetic fields at room temperature. The subfigure shows I–V curve of the junction between the two electrodes in absence of DNA molecules.

The I–V characteristic curve for gold-DNA-gold structure in the presence of various magnetic fields was measured in this setup (Figure 3). The characteristic curve I–V shows rectifying behavior that under forward bias, current increases exponentially with low threshold voltage. DNA strands, in this structure, act as a semiconductor equivalent to a back-to-back diode.

The potential barrier in this case is calculated using the metal-semiconductor contact equation according current-voltage-temperature and equates 0.878 eV approximately. Table 1 shows potential barrier (V_b_) and Richardson constant (A*) in different magnetic fields.

Table 1 lists variations of potential barrier obtained from I-V-T for application of magnetic fields with strengths less than 1,200.00 mT. Such variations are not considerable for field strengths of less than 1,000.00 mT, while an increase to 0.2886 eV is experienced for strength change of 800.00 to 1,200.00 mT.

**Figure 4.**
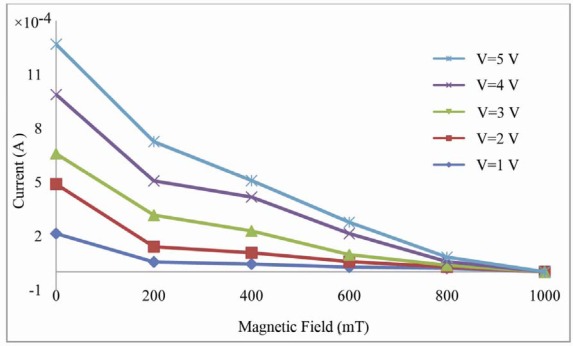
The magnitude of current in constant voltages of in several magnetic fields.

In other words, the effect of magnetic field (with strengths less than 1,000.00 mT) is not noticed at voltages lower than potential barrier and is realized as reduction of current for voltages higher than this value. For voltages less than potential barrier, the external field favors collisions of charge-carriers but the barrier confines them and therefore concentrated charges vary and deformation in the edge of energy band occurs. For voltages higher than potential barrier current was observed to drop with increase in exerted magnetic field as shown in Figure 4 for each constant voltage of 1, 2, 3, 4 and 5.0 V. With increasing magnitude of the magnetic field, the arrival time for carrier from the left to the right electrode will decrease and the current transfer rate will decrease.

**Figure 5.**
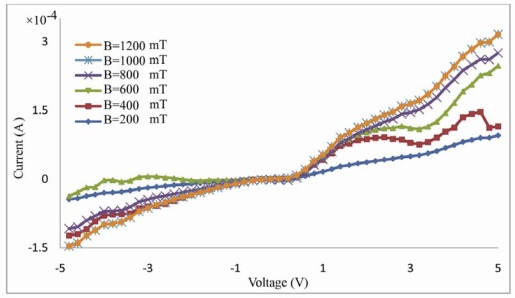
I(B=0)-I(B) via voltage in the presence of various magnetic field for gold-DNA-gold structure.

Once an external magnetic field is applied to the semiconductor, the energy levels increased allowing some of them to pass above the Fermi energy. In high magnetic field, the scattering amplitude for similar and dissimilar spins will differ [16] and the band gap will change according to discrete energy levels. In the presence of some low magnetic fields, the band gap does not change considerably. Also, the charge of material in a magnetic field will be subjected to a Lorentz force and Drude Theory. Electrons in partially filled bands and ions could be affected by the external magnetic field. A circular motion will be created with a trajectory of electron that is perpendicular to the field. Additionally, In Metal-DNA-Metal structures, before the charge carriers migrate from the left electrode to the right one, they move in a disturbed and spiral fashion to left and right for many times [14]. Such disturbed movements are due to the loose coupling of electrodes and DNA. In summary, an external magnetic field, alters the motion of carriers alongside the electric field and inhibits the reach of the carriers to the opposite electrode. This results in a decrease in current with respect to the case for absence of a magnetic field. To closer investigation of variations of current in present of magnetic field, differential current through MDM in presence and absence of magnetic field (I(B=0)-I(B)) *versus* voltage is illustrated in Figure 5. As seen, a gradual decline in current occurs with increase of field strength. It is known from this figure that in absence of magnetic field the current exceeds that of in its presence which results in a positive differential current in forward bias and negative in reverse. Therefore, the difference between the current in presence and absence of a magnetic field for greater magnetic fields increases and magnetic resistance rises with the field strength.

Such a behavior can be attributed to the increased collisions between charge-carriers and rotation of charges under the influence of external magnetic/electric fields and an increase in resistance. For a more detailed study on effective mechanism of current reduction, the saturation current *versus* magnetic field is calculated based on I–V analysis.

**Figure 6.**
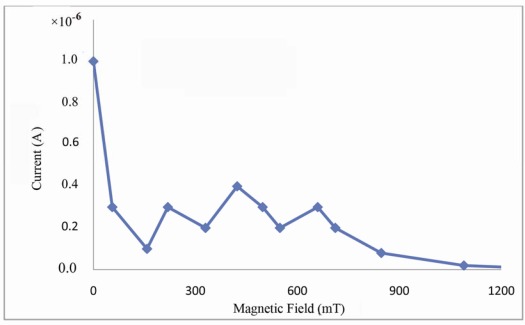
Saturation current *versus* magnetic field in gold-DNA-gold structure.

Figure 6 shows saturation current with respect to magnetic field strength. According to this figure, the saturation current undergoes a sharp decline and later on, oscillatory variations emerged.

An exponential decay in saturation current is observed with increase of field strength up to 1,000.00 mT. The initial decline obeys a linear relation and is due to decreasing mobility of carriers and decreasing A^*^ as well, as shown in Table 1, and the exponential change in saturation current is by the change in potential barrier height and the deformation of energy band in vicinity of gold-DNA interface.

However, due to its synergy with charge propagation near the barrier, the effect of image charge is lessened so that the barrier height obtained by I-V-T curves is more than the amount of splitting energy levels according to the Zeeman effect. Such an effect in reduction of carriers at high voltages is noticed as a decrease in carrier rates and an increase in resistance.

## 4. Conclusions

We report on the fabrication of a magnetic/nonmagnetic junction of DNA strands as a magnetic semiconductor/metal Schottky diode on a silicon dioxide substrate. In the absence of magnetic field, the MDM structure behaves like a conventional diode under applied voltage and also works like a magnetic diode under nonzero magnetic field. The I–V characteristics were measured in vertical MDM configuration perpendicular magnetic fields of 0 and 1,200.00 mT. Our findings ascertain that the Metal-DNA-Metal structure is capable of detection of magnetic fields with strengths equal and greater than 1 T. The dominant mechanism for field strengths less than 1 T is variation in mobility and propagation of carriers. While for larger strengths, orbital disintegration adds up. The magnetic sensitivity of gold-DNA-gold structure as illustrated by the graphs promotes DNA based devices as suitable candidate for magnetic field detectors/sensors and MDM structure makes a good option in fabrication of magnetic diodes and sensors.

## Figures and Tables

**Table 1.** Potential barrier via magnetic field in gold-DNA-gold structure (V_b_) and Richardson constant (A*) via magnetic field in gold-DNA-gold structure.

**B (mT)**	A* (Acm^−2^K^−2^)	**V_b_ (eV)**

0.00	110.079	0.878
200.00	129.454	0.880
400.00	135.014	0.882
600.00	187.596	0.890
800.00	204.640	0.886
1,000.00	254.186	1.157
1,200.00	298.586	1.175
